# Embryo competence and cryosurvival: Molecular and cellular features

**DOI:** 10.21451/1984-3143-AR2019-0072

**Published:** 2019-10-23

**Authors:** Thamiris V. Marsico, Janine de Camargo, Roniele S. Valente, Mateus J. Sudano

**Affiliations:** 1 Center for Natural and Human Sciences, Federal University of ABC, Santo André, SP, Brasil.; 2 School of Veterinary Medicine, Federal University of Pampa, Uruguaiana, RS, Brasil.

**Keywords:** *in vitro* production of embryos, bovine, embryo quality, cryopreservation, cryotolerance, pregnancy success

## Abstract

Global cattle genetic market is experiencing a change of strategy, large genetic companies, traditionally recognized in the artificial insemination field, have also begun to operate in the embryo market. Consequently, the demand for *in vitro* produced (IVP) embryos has grown. However, the overall efficiency of the biotechnology process remains low. Additionally, the lack of homogeneity of post-cryopreservation survival results of IVP embryos still impairing a massive dissemination of this biotechnology in the field. A great challenge for *in vitro* production labs is to increase the amount of embryos produced with exceptional quality after each round of *in vitro* fertilization. Herein, we discuss the molecular and cellular features associated with the competence and cryosurvival of IVP embryos. First, morphofunctional, cellular and molecular competence of the embryos were addressed and a relationship between embryo developmental ability and quality were established with cryosurvival and pregnancy success. Additionally, determinant factors of embryo competence and cryosurvival were discussed including the following effects: genotype, oocyte quality and follicular microenvironment, *in vitro* production conditions, and lipids and other determining molecules. Finally, embryo cryopreservation aspects were addressed and an embryo-focused approach to improve cryosurvival was presented.

## Introduction

**Figure 1:**
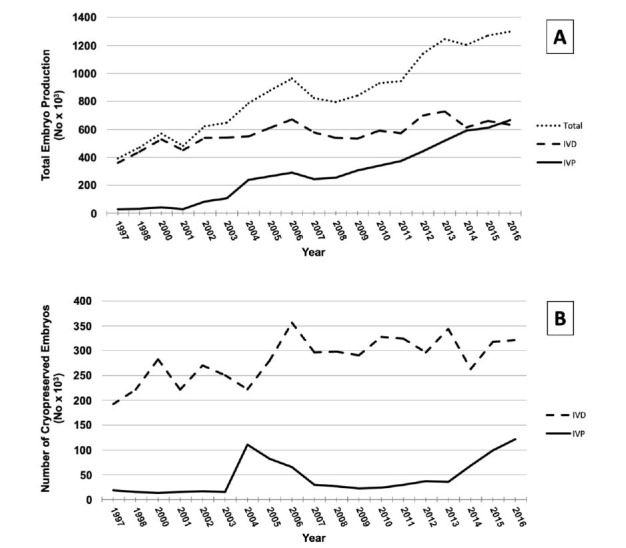
Total number, *in vivo* derived (IVD) and *in vitro* produced (IVP) bovine embryos in the world over the last 20 years (A). Number of cryopreserved IVD and IVP bovine embryos in the world in the last 20 years (B). Source: Data retrieval committee, International embryo technology society – IETS (https://www.iets.org/comm_data.asp?autotry=true&ULnotkn=true, accessed June 2019; and Perry, 2017).

In the last few years, the importance of *in vitro* production of embryos (IVPE) has increased within the reproductive biotechnologies applied in dairy and beef cattle. As a consequence, for the first time in the last two decades, the number of *in vitro* produced (IVP) surpassed the number of *in vivo* derived (IVD) embryos globally (Fig. 1A).

However, despite the favorable scenario associated with the IVPE, it is important to highlight that even with great effort of the scientific community aimed to improve embryo development and competence in the last decades, the overall efficiency of the biotechnology process remains low (Lonergan *et al*., 2016; Sudano *et al*., 2019).

Additionally, embryos derived from *in vitro* production process generally present a reduced quality compared with the *in vivo* counterparts (Rizos *et al*., 2002; Sudano *et al*., 2012). Consequently, even with important advances in the last years, the cryopreservation of IVP embryos remains as one of the most challenges areas of the embryo technologies since the homogeneity of the achieved results is still not satisfactory. These results impact directly in the reduced number of IVP embryos cryopreserved and transferred to recipients, in relation to the total transferred, when compared with the IVD embryos in the world over the last two decades (Fig. 1B).

Therefore, the objective of this work was to address: *i*) morphofunctional, cellular and molecular competence of embryos; *ii)* determinant factors of embryo competence and cryosurvival, including the following effects: genotype, oocyte quality and follicular microenvironment, *in vitro* production conditions, and lipids and other determining molecules; and *iii*) embryo cryopreservation aspects and an embryo-focused approach to improve cryosurvival.

## Embryo Competence

It is commonly accepted that *in vitro* produced (IVP) bovine embryos have lower developmental ability and quality than *in vivo*-derived (IVD) embryos (Hasler *et al*., 1995; Sudano *et al*., 2013a). The embryo developmental ability and quality are two crucial variables associated with cryosurvival, pregnancy establishment and maintenance that can be explained by morphofunctional, cellular and molecular competence of the embryos.

At the present review, the term embryo competence is used to describe the ability of the embryo to develop properly and with an exceptional quality that facilitates cryosurvival and/or pregnancy establishment.

### Embryo morphofunctional competence

Since the establishment of the biotechniques used in assisted reproduction in mammals, a good relationship between morphological evaluation of the embryo quality and pregnancy success after transfer has been established (Lindner and Wright, 1983; Overström, 1996). In most mammalian species, the morphological evaluation of the embryo is the most used method to select embryos suitable for transfer, mainly in cattle andhumans (Lindner and Wright, 1983; Van Soom *et al*., 2001). Currently the embryo evaluation system recommended by the International Embryo Technology Society (IETS) is described by Bó and Mapletoft (2013), which classifies the embryos into four quality rankings: (1) excellent or good, (2) regular, (3) bad or (4) dead or degenerate. Drastic changes in embryo’s morphology can directly affect its competence.

Assisted reproductive technologies such as *in vitro* production, nuclear transfer and gene transfer sheds light that imperceptible modifications in embryo structure can seriously compromise its quality. Two classical examples could be cited to illustrate this issue: the silicon/oil used to cover the microdrop culture affects the development of bovine embryos and their cryosurvival according to the product batch; however, embryos produced or not with this oil are morphologically similar. Another example is the parthenogenetic embryo, incapable of producing a successful pregnancy but impossible to be morphologic selected in the blastocyst stage from fertilized embryos (Van Soom *et al*., 2001).

Since embryos are mostly evaluated when it still confined in zona pellucida (ZP), this structure and the perivitelline space have some importance to discuss. The zona pellucida is a glycoprotein layer produced during oocyte growth phase surrounding the mammalian embryo between the zygote and the blastocyst stage. In average the thickness of ZP in mammals is 10 μm and its ultrastructure characteristics at oocytes (number and diameter of the pores) and embryos (thickness) can be associated with developmental competence (Santos *et al*., 2008; Báez *et al*., 2019) and hatching ability (Hoelker *et al*., 2006), respectively. It’s well known that, the ZP thickness decrease further the embryo development (Van Soom *et al*., 2003) and the average diameter of the ZP is significantly smaller at hatching for IVD versus IVP blastocysts (Holm *et al*., 2002). Although no relationship between ZP thickness and embryo viability after transfer to recipients was established (Hoelker et a., 2006), the ZP remains crucial for embryo handling before transfer to recipients in the majority of reproductive technologies, and especially, during embryo cryopreservation. Another parameter to be evaluated is the perivitelline space (PS), because it has been recognized that IVD embryos have larger PS than IVP embryos, possibly by the swelling of the blastomeres in the latter. The reduction in size of the PS could be associated with lower embryo quality and related with problems during the morula compaction stage (Van Soom *et al*., 2003).

Specifically, in the embryo itself, Merton (2002) pointed out some important variables to be considered in the global morphological evaluation, which included: size and shape of blastomeres, presence of extruded or fragmented cells, compaction, color and stage of development which is reached at a certain time after fertilization.

In comparison to humans and mice, all ruminants have a larger accumulation of lipid droplets in the embryo, which cause a quite dark cytoplasm, even darker in pigs, cats and dogs (Van Soom *et al*., 2003). This opacity disturbs the possibility of quantification of number of pronuclei at the zygote stages and thus select against triploids, parthenotes or unfertilized embryos an excellent method for selecting human embryos with high implantation potential (Lundqvist *et al*., 2001). Besides that, a polarized distribution of the pronuclei during pronuclear alignment is related to chromosomally normal embryos in humans (Coskun *et al*., 2003).

Studies show that the presence of serum in the culture medium can induce accumulation of cytoplasmic lipid droplets in bovine embryos. These fluctuations of cytoplasmic lipid droplets make darker embryos and lesser cryotolerant. The Sudan Black staining method has been vastly used for identifying embryos with increased numbers of lipid droplets (Abe *et al*., 2002; Sudano *et al*., 2011). Serum also affects the duration from maximal compaction to blastulation, shortening 12h in comparison to IVD embryos. This short compaction period and early blastulation in embryos coincided with perturbed allocation of cells to the inner cell mass and trophectoderm and is caused by decreased expression of transcripts that are involved in the construction of tight junctions, all this together decreased cryosurvival of the subsequently formed blastocysts (Van Soom *et al*., 1997; Miller *et al*., 2003).

Timing of blastocyst formation is a good marker for embryo quality as well early cavitating embryos are superior in comparison with the latter cavitating embryos in regard to total cell number, allocation of inner cell mass and trophectoderm cells, and cryosurvival (Mahmoudzadeh *et al*., 1995; Van Soom *et al*., 1997). Although, reliable morphological predictors at the blastocyst stage for competence after embryo transfer are still lacking. Nevertheless, there is a consensus by many research groups and commercial companies that the greatest results of pregnancy rates are generally achieved after the transfer of bovine day 7 expanded blastocysts regardless of whether they were fresh or cryopreserved (Hasler 2000; Xu *et al*., 2006; Block *et al*., 2009; Sanches *et al*., 2013; Munoz *et al*., 2014; Mogas, 2019).

Embryos suffer considerable morphofunctional damage when they are cryopreserved. The challenge during the process of vitrification/freezing followed by warming/thawing is enormous, which includes osmotic, thermic and mechanical stress. The extent of the cryopreservation injuries depends on factors such as the membrane permeability, size and shape of the cells, and quality and sensitivity of the blastomeres (Vajta and Kuwayama, 2006). In order to obtain greater cryosurvival results, therefore, it is sound to cryopreserve following transfer expanded blastocysts of extraordinary superior quality. No signs of degeneration in the morphological evaluation of embryos would be recommended (Hasler 2000; Xu *et al*., 2006; Block *et al*., 2009; Sanches *et al*., 2013; Munoz *et al*., 2014; Mogas, 2019).

### Embryo cellular competence

The initial development of embryos in mammals has unique physiological characteristics andmechanisms of regulation. Modifications in the properties of cytoplasmic organelles, such as location, morphology and biochemical activity must occur to achieve high quality during development (Sirard *et al*., 2006; Mao *et al*., 2014).

Initially, mitochondria constitute the powerhouses of cells, responsible for energy to boost all cellular functions. This organelle has an important role in calcium homoeostasis, fatty acid oxidation and apoptosis (Lledo *et al*., 2018). The embryo only starts to replicate it at hatched blastocyst stage, so all stages before depends on the mitochondrias pre-existent in the oocyte (Spikings *et al*., 2006). Alterations in normal mitochondrial functions can lead to metabolic diseases affecting the preimplantation development (Van Blerkom, 2004; Schaefer *et al*., 2008). In the early cell division cycle, the embryo has a greater amount of mitochondrias in storage, which is diluted as the divisions progress. This is the reason for blastocysts have few copies per cell, considered the mechanism for high quality transmission by the bottle neck theory (Lee *et al*., 2012). Therefore, just only at the implantation period the embryo is capable of replicate mitochondrial DNA (mtDNA) (Thouas *et al*., 2005; St John *et al*., 2010).

The initial production of energy in the early stages of embryonic development occurs through aerobic and anaerobic respiration (Thouas *et al*., 2005; El Shourbagy *et al*., 2006; Spikings *et al*., 2007), and is critical in several species. Human (May-Panloup *et al*., 2005), murine (Liu *et al*., 2000; Thouas *et al*., 2004), bovine (Chiaratti *et al*., 2011), and porcine (Spikings *et al*., 2007) studies have shown that problems in mitochondrial function lead to reduced cleavage rates and aneuploidy occurrence. In addition, low production of ATP and decrease of mtDNA copies are related with poor quality embryos (Wai *et al*., 2010; Wakefield *et al*., 2011). Another important change is the remodelling of mitochondrial features, turning into a complex structure that includes development of cristae, denser matrix and elongated form. These transitions are crucial to change from glycolytic to aerobic metabolism (Schatten *et al*., 2014).

Response to exogenous stress is a crucial part of cellular physiology. The main mechanism to start the cellular stress response pathway is located in the endoplasmic reticulum (ER). This organelle is responsible for secreting various proteins involved in several biological processes and its quality control is responsible for detecting failures to maintain the functioning of the cells which include processes of cell division, homeostasis, and differentiation (Latham, 2015). Disturbance in the endoplasmic reticulum stress signalling, especially in the GRP78 / BiP protein, leads to problems in the pre-implantation period, failure in blastocyst hatching and defects in cell division and apoptosis of the inner cell mass (Luo *et al*., 2006).

Another cellular component is the structure of the cytoskeleton, essential in diverse cellular functions and embryonic development. The cytoskeleton is composed of microfilaments, intermediate filaments and microtubules. Damage to the cytoskeleton adversely affects cell viability and can cause disruption in development and survival, given the support for intracellular content to be organized (Mao *et al*., 2014). In Damiman *et al* (2013) study, the comparison between slow-freezing and non-cryopreserved embryos showed a decrease of tubulin, actin and nucleus structures in each stage compared in mouse, indicating that may the vitrification technique would cause less damage on cytoskeleton (Dasiman *et al*., 2013). The IVPE can also modify the cytoskeleton leading, in some cases, to aberrant actin organization that is responsible for decreased development in IVP embryos (Mao *et al*., 2014). It was described in the literature that the supplementation of melatonin, a cytoskeletal modulator, reverse the damage in actin organization related genes, such as Rho/Rac guanine nucleotide exchange factor 2 (*Arhgef2*), BCL2 apoptosis regulator (*Bcl2*), coronin 2B (*Coro2b*), filamin C (*Flnc*), and palladin cytoskeletal associated protein (*Palld*), providing a target to optimize existing *in vitro* production systems (Tan *et al*., 2015).

Membrane damage and DNA fragmentation are the most commonly injury caused by cryopreservation (Sudano *et al*., 2012). Both injuries are associated with the reduction in the total cell number after cryopreservation and also the disturbance in the proportion of inner cell mass (ICM) / trophectoderm (TE) cells ratio (Sudano *et al*., 2012; Gomez, 2009). The proper ICM/TE ratio and cells number is crucial for embryo development and pregnancy establishment (Van Soom *et al*., 1996; Leese *et al*., 1998). These cryoinjuries can severe reduce embryo survival and the ability for the pregnancy success.

Many ultrastructural damages are commonly related to embryo cryopreservation, including: plasma membrane disruption, abnormal mitochondrial cristae, matrix swelling of the endoplasmic reticulum, poorly developed desmosome, reduced number of microvilli, disintegrations of cell adhesion among TE cells, and increase incidence of cell death (Overstrom *et al*., 1993; Vajta *et al*., 1997; Fair *et al*., 2001).

Nevertheless, many of this cryoinjury can be avoided by a proper cryopreservation methodology and rigorous application of technique assuring correct time of exposure and temperature of the cryopreservation steps. Additionally, cryopreserve exceptional quality embryos tend to reduce cryoinjuries, and clear signs of regeneration and reorganization of embryonic structures are expected after warming/thawing and re-culture, including re-establishment of tight-junctions between TE cells and the return of normal mitochondrial morphology (Vajta *et al*., 1997).

### Embryo molecular competence

Beyond morphological analyses, other techniques that reflect the functional and physiological state of the embryo have been proposed, such as the molecular analysis. Basically we can evaluate the embryos based into three groups of different stages of cellular functioning: (1) production of transcripts and proteins from specific genes of cellular products, (2) final products of cellular processes (metabolites) and (3)gene expression regulatory transcripts, the so-called small non-coding RNAs (ncRNAs), such as: interference RNA (siRNA), micro RNAs (miRNA) and antisense RNA (Phillips, 2008; Rodgaard *et al*., 2015). The present review focused on mRNA transcripts evaluation and discussed briefly other molecular aspects.

Morphological embryonic development events as first cleavages, compaction and blastulation are accompanied by a loss of pluripotency and formation of lineages of trophectoderm and inner cell mass to further development of the three primary germ layers: endoderm, mesoderm and ectoderm (Stephenson *et al*., 2012; Schrode *et al*., 2013). During this period, not only embryo morphology undergoes modifications, changes in the transcripts levels for genes associated with early embryo development dynamic occur. Transcription factors required for maintenance of pluripotency, such as POU class 5 homeobox 1 (*POU5F1/Oct4*), SRY-box transcription factor 2 (*SOX2*) and Nanog homeobox (*NANOG*), expressed strictly in ICM cells, mediated by the action of the caudal type homeobox 2 (*CDX2*) gene product expressed on trophectoderm cells (Chambers *et al*., 2003; Ralston and Rossant, 2008) have been widely described.

During the embryo pre-implantation period, there is a massive degradation of the maternal RNA/proteins stored inside the oocyte and the gradual activation of the embryonic genome. Studies that inhibit RNA polymerase II have demonstrated that the time of the embryonic genome activation (EGA) is related to the rate of embryonic development. In bovine, EGA occurs between 8-16 cells and is associated with early differentiation, embryo implantation success, and fetal development. However, the regulation of the gene expression of bovine embryos remains an unresolved biological issue (Misirlioglu *et al*., 2006; Graf *et al*., 2014).

A correlation between EGA with increased abundances of mature forms of miR30a and miR-21, and the primary form of miR-130a from 1-cell to 8-cell zygotes have been described (Mondou *et al*., 2012). Tripurani *et al* (2013) pointed to another miRNA with increased expression in 2- to 8-cell embryos, miR-212, possibly a suppressor of the maternal factor in the germ line at the transition of transcripts and maternal proteins to those of the embryo, called the maternal-embryonic transition. Although specific mechanisms of EGA are poorly defined, some factors involved such as maternal cyclin A2 (*CCNA2*), retinoblastoma protein (*RB1*), catalytic subunit of the SWI/SNF-related chromatin remodelling complex (*BRG1*), and the SRY-box transcription factor 2 (*SOX2*) have recently been suggested in an EGA model in mice (Tripurani *et al*., 2013). Embryos that do not properly undergo genome activation fail in the further development.

It has already been described that biopsies derived from IVP blastocysts that resulted in calf delivery were enriched with transcripts necessary for implantation (cytochrome c oxidase subunit II, *COX2* and caudal type homeobox 2, *CDX2*), carbohydrate metabolism (arachidonate 15-lipoxygenase, *ALOX15*), growth factor (bone morphogenetic protein 15, *BMP15*), signal transduction by plasminogen activator urokinase (plasminogen activator urokinase, *PLAU*) and placenta (placenta–specific 8, *PLAC8*). Transcripts involved in protein phosphorylation (keratin 8, *KRT8*), plasma membrane (occludin, *OCLN*) and glucose metabolism (phosphoglycerate kinase 1, *PGK1* and aldo-keto reductase family 1 member B1, *AKR1B1*) were enriched in reabsorbed embryos. Embryos that did not resulted in pregnancy presented enriched transcripts involved with inflammatory cytokines (tumor necrosis factor, *TNF*), amino acid binding protein (eukaryotic translation elongation factor 1 alpha 1, *EEF1A1*), transcription factors (msh bomeobox 1, *MSX1*, and PTTG1 regulator of sister chromatid separation securing, *PTTG1*), glucose metabolism (phosphoglycerate kinase 1, *PGK1* and aldo-keto reductase family 1 member B1, *AKR1B1*), and implantation inhibitor (CD9 molecule, *CD9*) (El-Sayed *et al*., 2006).

Despite great improvement of the information availability of embryo competence, this knowledge was not enough to avoid pregnancy loss observed in all mammalian species, in which cause serious issues such as reproductive wastage in farm animals. It is generally expected pregnancy loss in cattle in 40% of the cases between day 8 and 18 (Diskin and Sreenan, 1980). This embryo mortality is caused by a series of events that includes intrinsic embryo’s defects, unsatisfactory maternal environment and failure to synchronization between the cow and embryo.

Ghanem *et al*. (2011) identified 41 and 43 differently expressed genes between IVD blastocysts which resulted in no pregnancy *vs.* calf delivery and pregnancy loss *vs.* calf delivery, respectively. In general, genes related to placental development and maternal-embryo interaction (placenta–specific 8, *PLAC8*) were upregulated in embryos that had pregnancy to term. On the other hand, embryo’s biopsies that did not end in successful pregnancy presented enriched transcripts related to mitochondrial transcripts (*Bos taurus* isolate FL405 mitochondrion, *Fl405*) and stress genes (heat shock protein family D (Hsp60) member 1, *HSPD1).* However, both biopsies presented similar gene expression related to preimplantation development of embryo.

Another study also evaluating the molecular signatures of IVD embryo biopsies which resulted in no pregnancy and calf delivery identified 70 differently expressed genes: 32 transcripts levels were upregulated in the biopsies from calf delivery-derived embryos, such as sperm associated antigen 17 (*SPAG17/PF6*), ubiquitin conjugating enzyme E2 D3 (*UBE2D3P*), deafness autosomal recessive 31 (*DFNB31*), S-adenosylmethionine decarboxylase 1 (*AMD1*), dystrobrevin binding protein 1 (*DTNBP1*), and adp-ribosylation factor-like 8B (*ARL8B*); whereas 38 transcripts levels including RING1 and YY1 binding protein (*RYBP*), ring finger protein 34 (*RNF34*), karyopherin alpha 4 (*KPNA4*), and WD repeat domain 13 (*WDR13*) were increased in the biopsies of the no pregnancy-derived embryos indicating that the embryos are molecularly distinguishable (Salilew-Wondim *et al*., 2010). The biopsy transcriptional profiles from cryopreserved blastocysts that resulted in calf delivery, no pregnancy, and pregnancy loss are still lacking.

Our group have recently evaluated the transcriptional profiles of IVP bovine embryos with high and low post-cryopreservation survival. Blastocysts with high cryosurvival were enriched in 27 genes associated with the following top five biological processes: a) predicted to be activated: organismal survival, cell death and survival, cellular growth and proliferation; and b) predicted to be inhibited: cellular movement and cell-to-cell signalling (data not published).

A favourable transcriptional profile for pregnancy establishment and maintenance, therefore, could be used as a marker of embryo competence. In addition, each gene and/or genes involved pathway should be studied in order to design strategies applied to the *in vitro* embryo production system to improve embryo quality, cryosurvival and embryo-derived pregnancy.

## Determinant effects of embryo competence and cryosurvival

Early embryo development period is complex, conserved, and well-orchestrated process involving dynamic molecular and structures changes. Embryos must undergo important events, such as fertilization, cleavage, epigenetic reprogramming, compaction, differentiation, and blastulation, for proper development and pregnancy establishment (Sudano *et al*., 2016a; Jiang *et al*., 2014).

**Figure 2:**
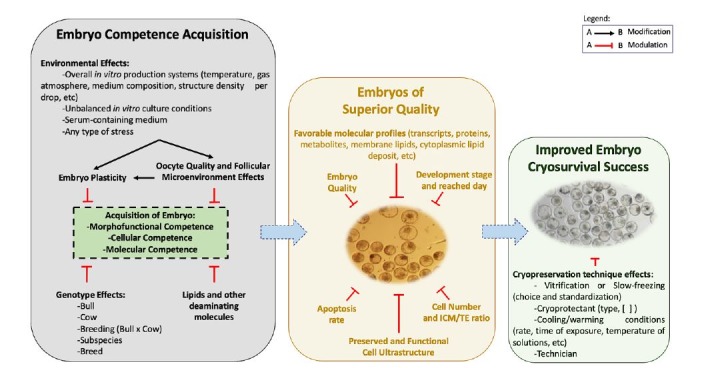
Cellular and molecular events required for embryonic developmental competence acquisition and its impact on cryosurvival. Interconnected steps (blue dashed arrows, from the left to the right) to *in vitro* produce an embryo of superior quality and improved embryo cryosurvival success. Overall environmental and genetic effects on embryo competence acquisition (Black Box). Determinant variables associated with the *in vitro* production of superior embryo quality (Gold Box, in detail fresh IVP blastocysts with great quality). Successful resumption of development after cryopreservation and cryopreservation technique effects (Green Box, in detail re-expanded IVP blastocysts after warming). All Box events (Black, Gold and Green) impact embryo cryosurvival.

Several factors can disrupt competence during this period and result in variations of the embryo phenotypes, including genotype and environmental effects. Because of this reason, a rigorous quality control should be used to perform all the steps involved in the IVPE in order to mimic as much as possible the *in vivo* environment conditions and reduce possible genotype effect. A putative model addressing cellular and molecular events required for acquisition of embryonic developmental competence and cryosurvival is presented at Figure 2.

### Genotype effect

It is well documented in the literature the great variation in the developmental competence of IVP bovine embryos according to the sire (Brackett and Zuelke, 1993; Galli and Lazzari, 1996). Variables like cleavage and blastocyst production rates of a specific bull and/or semen batch (with unknow IVF results) are generally previously evaluated *in vitro* before the vastly use of this semen in the IVPE routine. Nevertheless, there are considerable variations of pregnancy rates observed after the embryo transfer according to the sire used for both fresh and cryopreserved embryos.

Our group recently evaluated the sire effect in the pregnancy rates following the transfer of fresh (N = 40,200) or cryopreserved (N = 9,858) IVP embryos. Pregnancy rates varying among 28.3 to 52.5% (for fresh) and 7.7 to 61.6% (for cryopreserved) were recorded after transfer IVP embryos derived from different bulls. We also have observed a variation according the cow, breeding, subspecies, and breed on pregnancy rates (Sudano *et al*., 2019). However, despite great amount of the variation was attributed to the genotype effect in this evaluation, it is also important to considerate the environmental effect in the determination of the reason of variation.

Variations in the membrane lipid profiles of phosphatidylcholine and sphingomyelin between *Bos taurus indicus* (Nellore) and *Bos taurus taurus* (Simmental) embryos and its impact on the embryo’s cryosurvival have already been reported (Sudano *et al*., 2012; 2013a). After vitrification the evaluation of the percentage of structures hatching revealed results superior to *B. taurus taurus* (34.6%) compared to *B. taurus indicus* (20.2%) embryos (Sudano *et al*., 2013a). Arreseigor *et al*. (1998) also showed this difference between subspecies in the pregnancy rate. It seems that factors including the phospholipid cell membrane composition are associated with the determination of the physicochemical properties such as fluidity, permeability and thermal phase behavior, which are determinants for cryotolerance (Van Meer *et al*., 2008).

### Oocyte quality and the follicular environment effects

The production of competent oocytes is dependent on complete nuclear and cytoplasmic maturation. The oocyte during its development undergoes rearrangements that make it capable of supporting fertilization and early embryonic development. During the nuclear maturation process, oocyte chromosomes go through diplotene of prophase I to metaphase II of meiosis. Among the main changes in cytoplasmic maturation are carbohydrate and lipid metabolism, mitochondrial function and location, reduction of oxygen radicals, epigenetic reprogramming, bidirectional communication between the cumulus-oocyte complexes (COC) and its secretion of growth factors (Brevini Gandolfi and Gandolfi, 2001). All this events are crucial for a proper oocyte competence acquisition.

In the commercial IVPE programs, the ultrasound-guided follicular aspiration (OPU) is routinely used for oocyte recovery, this biotechnology allows access a large number of female gametes inmono-ovulatory species favoring the multiplication of animals with exponentially genetic merit (Nagai *et al*., 2015; Nagano, 2019). Investigations on the influence of the follicle microenvironment and size in the establishment of competent embryos have been conducted (Annes *et al*., 2018; Labrecque *et al*., 2016; Lonergan *et al*., 2016).

There is considerable effect of follicle conditions in the oocyte and embryo competence. Oocyte origin is the main factor affecting blastocyst yield (Rizos *et al*., 2002). Any type of stress conditions that oocytes face can potentially disrupts embryo development. Differences in the morphology (Nagai *et al*., 2015; Nagano, 2019), lipid composition, transcriptome, and embryo development (Labrecque *et al*., 2016; Annes *et al*., 2018) had already been described in the oocytes recovery from different follicle sizes. The bovine oocytes used for IVPE are recovered from follicles with 2 to 8 mm of diameter, before follicle divergence. Generally, oocytes recovered from large follicles (> 6mm) present a greater potential of reaching blastocysts compared to oocytes recovered from follicles of 2 to 6 mm (Rizos *et al*., 2002; Fair, 2003; Labrecque *et al*., 2016; Annes *et al*, 2018).

Despite embryo culture conditions is crucial for embryo quality and cryosurvival determination (Rizos *et al*., 2002), blastocysts that originate from oocytes matured *in vitro* result in lower rates of gestation compared to their *in vivo* counterparts (Peterson and Lee, 2003). Additionally, we recently identified a relationship between the cytoplasmic lipid content of oocytes and the lipid deposit of expanded blastocysts (Annes *et al*., 2018), i.e., oocytes derived from large follicles and containing greater amount of lipid droplets, originated day 7 expanded blastocysts, after IVPE, with increased lipid deposit. An experiment evaluating the cryosurvival of these blastocysts is still lacking, however, it is fair to speculate that this increased lipid deposit negatively impact embryo cryosurvival (Abe *et al*., 2002).

This results collaborate in the selection of follicles/oocytes of greater potential, since key events for embryonic development are dependent of oocyte status (Fair, 2010). A properly embryo developmental competence, therefore, could be increased in an oocyte focused manner.

### In vitro production conditions effects

It is commonly accepted that IVP embryos have a reduced competence compared with the *in vivo* counterparts. *In vitro* production of embryos is a three-step procedure involving oocyte maturation, fertilization, and *in vitro* culture of embryos. The *in vitro* environment directly influences the embryonic phenotypes and results. It is very important, therefore, to mimic the conditions found *in vivo* in order to allow all events inherent to the early embryonic development occur (for review, see Sudano *et al*., 2012; 2013a; 2013b).

Since the first notice of a calf delivery from a completely IVP embryo (*in vitro* maturation, fertilization and culture; Lu *et al*., 1987) the *in vitro* production systems still have challenging outcomes. Despite 80 to 90% of oocytes submitted to *in vitro* maturation have the germinal vesicle breaks down from prophase I to metaphase II, and 80% cleave after fertilization, only 20 to 40% of the oocytes develop to the blastocyst stage, and 50% of transferred IVP blastocysts establish pregnancy, i.e., just only 10 to 20% of the recovered oocytes submitted to *in vitro* production will result in pregnancy (Lonergan *et al*., 2016, 2006; Rizos *et al*., 2008; Wrenzycki and Stinshoff, 2013; Sudano *et al*., 2019).

*In vivo*, oocyte maturation occurs during follicle development, a period that oocytes produce and storage mRNAs and protein molecules necessary to supply the first embryonic activities (Brevini Gandolfi and Gandolfi, 2001). *In vitro*, the maturation medium and *in vitro* process are responsible to provide conditions to the oocytes undergo nuclear (reach metaphase II of meiosis), cytoplasmic (re-distribution of organelles such as the mitochondria and the cortical granules) and molecular (accumulation of specific molecules, largely unidentified, which prepare the oocyte for post-fertilization events) maturation (Sirard *et al*., 2006). However, the *in vitro* maturation conditions are still not so much efficient. It was reported that blastocyst production from oocytes matured *in vitro* is lower than *in vivo* (39.2% vs. 58.2%, respectively; Rizos *et al*., 2002). Even if different supplements like grown factors and hormones were supplemented during IVM, the blastocyst development rate was not higher compared with the serum-supplemented medium highlighting the importance of the initial status of the oocyte (Ward *et al*., 2002; Hoelker *et al*., 2014).

In this context, there are some investigative approach studies describing a greater gene expression pattern correspondent to cytoplasmic maturation involved genes in oocytes with higher blastocyst production (Räty *et al*., 2011) and also a greater expression for apoptosis activating related genes to non-competent structures (Warzych *et al*., 2007). This kind of works and a better understanding of *in vivo* oocyte maturation could improve the development of the maturation medium that produces viable embryos (Wrenzycki and Stinshoff, 2013).

In the literature, there is a consensus that improvements in oocyte quality is crucial for the proportion of oocytes developing to blastocysts stage, whereas the post-fertilization environment period is the major aspect determining the blastocyst quality and competence, including post-cryopreservation survival (Rizos *et al*., 2002; Lonergan *et al*., 2003; Hoelker *et al*., 2014).

*In vivo*, fertilization and early embryonic development take place at the oviduct due to the ability of this microenvironment to support embryogenesis, providing nutrients, growth factors, antioxidants, sexhormones, proteases, and other regulatory molecules of gametes and embryos. Furthermore, the cellular structure of this reproductive organ allows the transport of embryos to the uterus (Li and Winuthayanon, 2017; Rizos *et al*., 2008, 2002). Mimic *in vivo* environment is very difficult and somehow we generally failed in this task. Unbalanced *in vitro* culture conditions itself impacts directly embryo competence, since it has a significant effect on the cellular metabolism (D’Souza *et al*., 2018; Khurana and Niemann, 2000; Rizos *et al*., 2002) quality and quantity of lipids (Sudano *et al*., 2016a), gene expression patterns (Lonergan *et al*., 2006), and on modifications in epigenetic markers, which can continue after birth (Ramos-Ibeas *et al*., 2019).

However, despite the great diversity of culture media and systems available and used for IVPE with research purpose and commercially, they generally are associated with a relative good developmental potential which sheds light to the embryo plasticity, i.e., the capacity to fetch survival and adapt to adverse conditions even in an environment that do not supply or exacerbate their physiological needs (Khurana and Niemann, 2000; Lonergan *et al*., 2006). For example, the capacity of switching energy consumption source according nutrient availability. In mice, this plasticity was represented by the significant increase of the consumption of pyruvate, due to lack of glucose in the used medium (Gardner and Leese, 1988).

At molecular level, the great plasticity and tolerance to distinct culture conditions/systems including temperature, gas atmosphere, medium composition, embryo density per drop, and many other situations, are associated with an exacerbate gene expression pattern on IVP embryos compared with the *in vivo* counterparts (Corcoran *et al*., 2006; Côté *et al*., 2011; Sudano *et al*., 2013a).

However, this embryo plasticity can cause some severe damage to its competence and offspring; e.g. the large offspring syndrome in cattle (McEvoy *et al*., 2000; Rizos *et al*., 2008) and the overweighting newborns in humans (Li and Winuthayanon, 2017). In bovines, this anomaly is associated with a protein source used in the culture media, the fetal calf serum (FCS), characterized by abnormal fetal and placental size, increased myogenesis, dystocia, abnormal neonatal lung activity, and increased post-neonatal mortality (McEvoy *et al*., 2000; Walker *et al*., 1996). In addition, as previously cited in this review, the FCS supplementation was also associated with increased cytoplasmic lipid deposit and reduced cryosurvival of the embryos (Abe *et al*., 2002; Sudano., 2011). In a previous work of our group, the cytoplasmic lipid droplets fluctuation observed on IVP embryos was closed related with FCS concentration supplemented to the culture media (Sudano *et al*., 2011) and with the mRNA levels for *ACSL3* (Sudano *et al*. 2016a).

Taking all these studies together, culture conditions dramatically affect embryo developmental potential, quality, and further ability to survive after cryopreservation, and establish and maintain pregnancy. Despite many knowledge of the *in vitro* production systems have been produced over the past decades, efficiency improvements in the biotechnology process of the bovine IVPE is still needed in order to increase blastocyst yield of an exceptional quality.

### Lipids and other determining molecules for embryo competence

The morphological transitions performed by embryos during the early development until implantation are accompanied by changes in the substrate requirements due to the embryo metabolism demand during this period. *In vivo*, at the reproductive tract, where embryo development takes place, sources of amino acids, proteins, lactate, pyruvate, glucose, antioxidants, ions, growth factors, hormones, and lipids are dynamically available with variations in concentration according to species, estrous cycle period and location (Di Paolo and De Camilli, 2006).

The measurement of embryonic metabolism can be used as an important tool in the analysis of molecular and cellular competence (Thompson *et al*., 2016). Hamatani *et al*. (2004) described two different physiological profiles of blastocysts (dormancy and activation) according to the global expression of genes involved in cell cycle control, cell signaling, and energy metabolism. In a total of 18 genes associated with metabolism, 13 were expressed in blastocysts on activation and 5 in blastocysts on dormancy. These metabolism variations contributed to the further hypothesis of “quiet embryos”, which describes the metabolic efficiency of competent embryos with the lower need of substrate for development as a result of a lower percentage of cell damage based in the comparison of IVD and IVP embryos (Leese *et al*., 2008, 2007).

When analyzing the energy metabolism during early embryonic development, energy consumption is relatively low until pre-compaction stage and the main source of ATP is derived from pyruvate through oxidative phosphorylation, whereas with blastocyst formation and cavitation process a significant increase on energy demand and consumption of glucose, pyruvate and oxygen are expected. Additionally, after formation of the blastocyst, other sources of energy are required in greater proportion, including: glucose, amino acids and lipids (D’Souza *et al*., 2018; Hu and Yu, 2017).

Because of the various formulations of medium used by IVF laboratories, the understanding of metabolic spent during IVPE helps in directing compositions that structure viable embryos to the establishment of pregnancy (Thompson *et al*., 2016). Recent studies showed a higher energetic requirement of pyruvate and lactate, during culture, for non-competent embryos (D’Souza *et al*., 2018). The influence of the culture medium on the embryonicdevelopment and the production of healthy animals is already well established, so research that identifies the potential of energy supplements used in the bovine IVPE prevents the multiplication of inappropriate phenotypes (De Souza *et al*., 2015).

Additionally, lipids are an important source of ATP for cell development and actively participates in embryonic metabolic pathways (Hu and Yu, 2017). Lipids are responsible for the cross-talk between embryo, oviduct and uterus. This event is necessary in the synchronization of molecular and cellular events during the pregnancy establishment.

Studies demonstrated the lipid importance in signaling and coordination of biological events by mediating lipids such as phosphatidylinositols, sphingolipids, and eicosanoids (Di Paolo and De Camilli, 2006; Wang and Dey, 2005). Lipids are essential cellular biomolecules for plasma membrane and membranes of several organelles (Di Paolo and De Camilli, 2006). In bovine embryos, a stage-specific dynamic lipid fluctuation was observed during early embryo development (Sudano *et al*., 2016a). In addition, our group identified an increase in the mRNA levels for *ELOVL5* and *ELOVL6* (two transcripts of the ELOVLs family, responsible for fatty acids elongation) at the morula stage preceding an increase in membrane phospholipids containing elongated saturated, monosaturated and polysaturated fatty acids (16, 18 and 20 carbons) at the blastocyst stage (Sudano *et al*., 2016a).

Cells can synthesize simple fatty acids due to their biological functions. In this sense, lipid elongation facilitates the formation of complex biomolecules for specific activities (Guillou *et al*., 2010). We have recently observed the involvement of the ELOVL5 in the lipid metabolism of IVP embryos by regulating the cytoplasmic lipid deposit and the abundance of phosphatidylcholines, phosphatidylethanolamines and triacylglycerol (data not published).

All these findings demonstrate the importance of understanding the mechanisms involved in energy metabolism and embryo lipids composition in order to optimize *in vitro* culture conditions to allow the production of embryos with superior quality and greater cryosurvival.

## Embryo cryopreservation

**Figure 3:**
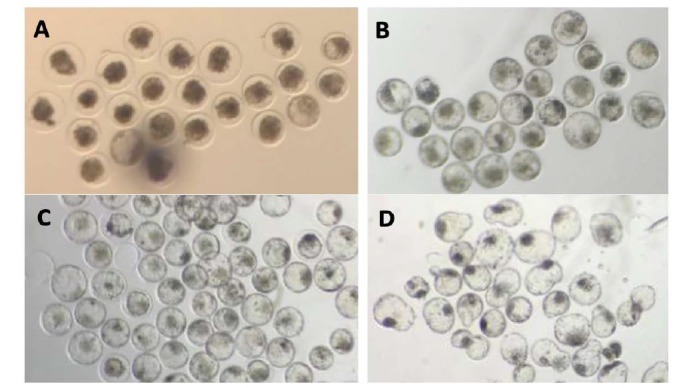
Vitrified *in vitro* produced bovine embryos immediately after warming (A), and following 6 h (B) and 12 h (re-expanded and hatched blastocysts, respectively for C and D) of re-culture.

Embryo cryopreservation is a nitrogen (N_2_) based conservation biotechnology that aims to maintain the cells in quiescent state, prolong the viability for long periods of time and enable their use in a timely manner (Fig. 3 3). In the area of assisted reproduction, primordial germ cells, gametes and embryos can be cryopreserved. It is considered a strategy to overcome logistical problems associated with the transfer of a large number of fresh embryos and mainly for the expansion of the commercialization of embryos between countries (Sudano *et al*., 2013b). Among the advantages are the optimization of reproductive biotechnologies such as IVPE and transfer of embryos in order to preserve surplus embryos, conservation of the genetic material of endangered species, prevention of problems arising from the transport of live animals and adaptations of the calving season.

The first success report on the survival of embryos (mice) subjected to freezing/thawing was recorded by Whittingham *et al*. (1972). In the following years, the technique was also applied successfully in different species and embryo cryopreservation started to be applied commercially. In Brazil, the majority of the IVP embryos are still transferred fresh, but the percentage of frozen (33.7%) follows a growth trend, and among the embryos produced in *vivo*, the slow freezing is the preferred option (62.1% of the total) (Viana, 2018). The principle of cryopreservation consists of maximum removal of water from the interior of the cells before freezing, avoiding the formation of large ice crystals that cause cell damage, which allows the resumption of cellular metabolism after storage at low temperatures. For this reason, cryopreservation strategies are based on two main factors: cooling/warming rates and choice of suitable cryoprotectants (CPA) (Vajta and Kuwayama, 2006).

According to Mazur (1980) there is a correlation between the cooling rate and the appearance of cell lesions, i.e. as the freezing rate increases, the survival after thawing also increases, until an ideal maximum index is reached from which the higher freezing speed also leads to greater damage and lower embryo survival. In addition to the freezing rate curve, the use of CPA is essential to provide cellular protection against temperature drop. Although CPA are absolutely necessary and widely used in cryopreservation protocols, the mechanisms that confer protection of the biological material as well as the toxicity and cellular metabolism of these agents are not fully elucidated and addressed in the literature (Castro *et al*., 2011). For review all cryobiology principles involved on embryo cryopreservation, please see Sudano *et al*. (2016b).

It is known, at least partially, that the CPA protective effect comes from the reduction of the solidification point of the solutions and the ability on intermediating intracellular water removal/restauration. According to their ability to penetrate cell membranes, CPA are divided into two groups: permeable (e.g. glycerol, ethylene glycol and propylene glycol) and impermeable (e.g. sucrose, galactose, polyethylene glycol and polyvinylpyrrolidone). In addition, there are two major cryopreservation methodology currently applied for bovine embryos, slow freezing and vitrification.

### Cryopreservation techniques

Slow or classic freezing is one of the most widespread embryo cryopreservation techniques and is based on the use of glycerol or ethylene glycol as a CPA. The main advantage of this technique is the reduced cellular toxicity through the use of low concentrations of CPA, however, slow freezing still allows the formation of ice crystals that could lead to cellular damage. After a period of previous CPA exposure, the embryos are loaded in straws together with the CPA solution, which will be allocated in programmable freezing devices for cooling. The temperature drop is controlled by keeping a constant curve until the temperature reaches -32°C, when the straws containing the embryos are immersed in the liquid nitrogen. After thawing, the embryos must be placed in petri dishes where the CPA solution will be diluted by successive washes in dilution solutions. Alternatively, Leibo (1983) developed the One-Step method where dilution of the CPA still occurs in the straw after thawing, letting the transfer to recipients be without previous microscopic evaluation.

Although slow freezing is the most widely used method for the cryopreservation of IVD embryos, vitrification has emerged as an alternative to IVP embryos. When comparing the cryosurvival and pregnancy results of both procedures considering the production method, IVD embryos present similar results, for both slow freezing and vitrification, whereas IVP embryos present more satisfactory results with vitrification (Tab. 1 and 2). However, slow-freezing also can be a good option to cryopreserve IVP embryos, Sanches *et al*. (2016) reported similar pregnancy rates on Day 60 after the transfer of IVP embryo cryopreserved by slow-freezing (34.7%, n = 311) or vitrification (31.2%, n = 234).

**Table 1:** Post-cryopreservation survival of *in vitro* produced bovine embryos following slow-freezing and vitrification.

Reference		Slow-freezing (%)			Vitrification (%)			P-value
		Re-expansion	Hatching		Re-expansion	Hatching		
Dinnyés *et al*., 1996		62.0^a^ (n = 63)	81.0^A^ (n=63)		81.0^b^ (n = 64)	70.0^A^ (n = 64)		<or>0.05
O’Kearney-Flynn *et al*., 1998		58^a^ (n =73)	-		86^b^ (n = 64)	-		<0.05
Nedambale *et al*., 2004		40.0^a^ (n = 297)	22.0^A^ (n = 297)		64^b^ (n = 297)	54.0^B^ (n = 297)		<0.05
Mucci *et al*., 2006		16.7^a^ (n = 275)	19.6^A^ (n = 275)		52.1^b^ (n = 265)	51.3^B^ (n = 265)		<0.05
Barceló-Fimbres and Seidel, 2007		80.4 (n = 360)	19.1 (n = 360)		77.7 (n = 360)	16.0 (n = 360)		>0.05
Yu *et al*., 2010		46.9^a^ (n = 155)	24.7^A^ (n = 155)		58^b^ (n = 153)	36.2^B^ (n = 153)		<0.05
Inaba *et al*., 2011		88.6 (n = 44)	75.0^A^ (n = 44)		100 (n = 44)	93.2^B^ (n = 44)		<0.05

Re-expansion rate was evaluated 24h after thawing/warming and hatching/hatched rate was evaluate with 48 or 72 h after thawing/warming according the study methodology. Values without a common lowercase (comparisons between re-expansion rates) or uppercase (comparisons between hatching rates) letters differ (P < 0.05).

**Table 2:** Post-cryopreservation survival of *in vivo* produced bovine embryos following slow-freezing and vitrification.

Reference		Pregnancy rate (%)			P-value
		Slow-freezing	Vitrification		
Massip *et al*., 1987		51.8 (n = 27)	39.1 (n = 23)		>0.05
van Wagtendonk-de Leeuw *et al*., 1995		59 (n = 40)	43 (n = 34)		0.10
van Wagtendonk-de Leeuw *et al*., 1997		45.1 (n = 335)	44.5 (n = 393)		0.79
Mattos *et al*., 2010		19.5 (n = 79)	17.8 (n = 73)		>0.05
		29.8 (n = 102)	36.6 (n = 100)		>0.05
Inaba *et al*., 2011		45.2 (n = 62)	46.7 (n = 30)		>0.05

Vitrification eradicates the damage caused by the formation of ice crystals during the cooling process. The cryopreservation medium undergoes a direct passage from the liquid state to a vitrified and amorphous state without the crystallization of the medium occurring, which it is possible due to the high viscosity of the cryopreservation medium and the high freezing rate (>20.000°C/min) by direct immersion in N_2_, from room temperature (Fahy *et al*., 1984). The technique is extremely fast and does not require the use of freezing devices; however, it requires more training and technical skills from the technician. Among the disadvantages are the difficulty of direct transfer and the need to use high concentrations of CPA that can cause cellular toxicity. As a result, several combinations with different CPA, concentrations and period of exposure have already been proposed. In order to ensure a rapid cooling rate, the use of the minimum volume of solution per sample is adopted in order to provide immediate contact with the N_2_. For this purpose, specific tools have been developed for packaging the embryos into microdrops, such as *Open Pulled Straw* (OPS) (Vajta *et al*., 1998), *Cryoloop* (Lane *et al*., 1999) and *Cryotop* (Kuwayama, 2007). More recently, a new automated vitrification methodology has been proposed (Arav *et al*., 2018), which may facilitate and optimize the standardization of the technique.

Despite great results were achieved with enhancements of the cryopreservation methodology over the last decades; an embryo-focused approach to improve cryosurvival has been recommended.

### An embryo-focused approach to improve cryosurvival

The effect of cryopreservation on mammalian embryos reduces survival rates, leading to considerable morphological and functional damage. However, the extent of cryoinjury is highly variable and dependent on the species, stage of development and origin of the embryo (produced *in vivo* or *in vitro*) at the time of cryopreservation (Dalcin and Lucci, 2010).

There is a consensus in the literature that IVP embryos present lesser cryoresistance compared with IVD embryos. Ultrastructural and biochemical characteristics such as increased lipid content, reduced intercellular junctions, fewer mitochondria and microvilli, larger perivitelline space and more cell debris are generally associated with the reduced cryosurvival of IVP embryos (Fair *et al*., 2001; Abe *et al*., 2002, Rizos *et al*. 2002). Additional variables associated with embryo cryosurvival are presented on Table 3. Thus, some strategies can be used to increase the cryotolerance of IVP embryos, such as culture under a low oxygen atmosphere system to minimize oxidative stress, addition of antioxidants to the culture medium, and the use of apoptosis inhibitors (Lin *et al*., 2018; Pero *et al*., 2018).

**Table 3:** Features positively or negatively associated with cryosurvival evaluated so far by our group.

Variables	Associated Cryosurvival
Sire	Positively or Negatively
Genotype (*Bos Taurus taurus vs. Bos Taurus indicus*)	Positively or Negatively
Metabolic regulators and lipolytic molecule	Positively, Negatively or none
Embryo origin (IVP *vs.* IVD)	Positively or Negatively
Increased embryo quality	Positively
Increased fresh apoptosis rate	Negatively
Increased cytoplasmic lipid content	Negatively or positively
Membrane phospholipids profiles	Negatively or positively
Increased serum concentration	Negatively

As already mentioned, excessive lipid droplets accumulation of IVP embryos during development, especially of embryos cultured in serum-supplemented medium, is commonly associated with reduced cryosurvival and lower pregnancy rate (Rizos *et al*., 2008; Sudano *et al*., 2011). The exact mechanism for this increased lipid content on IVP embryos remains unknown. It seems that serum lipids could be absorbed by embryonic cells (Sata *et al*., 1999), altering the function of mitochondrial β-oxidation (Abe *et al*., 2002) and promoting the incorporation of saturated fatty acids and cholesterol in the cell membranes, which make them less permeable (Barceló-Fimbres and Seidel Jr, 2007). All these factors together also could explain the greater susceptibility of the IVP embryos to cryopreservation.

In fact, it is possible to produce embryos with a greater cryosurvival through manipulations of the culture medium. We have already demonstrated that only the reduction of the serum concentration in the developmental medium was capable of decrease thelipid content and increase embryo survival after cryopreservation (Sudano *et al*., 2011). Recently, our group participate in the development of a serum-free culture medium of IVP embryos that reach similar results of embryo quality and pregnancy rate compared with a serum-supplemented medium (data not published).

Alternatively, chemical substances that act as a delipidant or lipolytic agents, such as Forskolin (Sanches *et al*., 2013; Paschoal *et al*., 2014), L-carnitine (Held-Hoelker *et al*., 2017), and 10t,12c-CLA (Pereira *et al*., 2007) have been commonly adopted in the attempt to reduce the embryonic lipid content and improve cryosurvival. In addition, embryo delipidation after centrifugation and subsequent micromanipulation has already been proposed (Ushijima *et al*., 1999), however, considering this is an invasive and time-consuming technique, this approach had become impracticable for large scale application and was limited for scientific purpose.

An additional strategy to improve cryosurvival of IVP embryos is the preincubation with grow-stimulating factors before cryopreservation. Beneficial effects of insulin-like growth factor 1 and leukaemia inhibitory factor were observed in the post-cryopreservation survival of bovine blastocysts (Kocyigit and Cevik, 2015).

## Final considerations

Embryo developmental competence and quality are crucial for cryosurvival and pregnancy success. Many variables can impair embryo competence and cryosurvival such as genotype, oocyte quality and follicular microenvironment, *in vitro* production conditions, and lipids and other determining molecules. A great quality control of all steps of IPVE and the use of exceptional quality embryos are recommended to improve and achieve homogenous results. A promising scenario for the next few years is expected for the use of the IVP embryos in the reproductive management of beef and dairy cattle. However, it is essential that further research efforts focus on improvements of the efficiency of producing a greater amount of embryos of superior quality, per oocytes recovered and round of *in vitro* fertilization, to be cryopreserved following transferred in order to reduce costs and justify the activity economically.
